# Genome-wide analysis of T-DNA integration into the chromosomes of *Magnaporthe oryzae*

**DOI:** 10.1111/j.1365-2958.2007.05918.x

**Published:** 2007-10

**Authors:** Jaehyuk Choi, Jongsun Park, Junhyun Jeon, Myoung-Hwan Chi, Jaeduk Goh, Sung-Yong Yoo, Jaejin Park, Kyongyong Jung, Hyojeong Kim, Sook-Young Park, Hee-Sool Rho, Soonok Kim, Byeong Ryun Kim, Seong-Sook Han, Seogchan Kang, Yong-Hwan Lee

**Affiliations:** 1 Department of Agricultural Biotechnology, Center for Fungal Genetic Resources, and Center for Agricultural Biomaterials, Seoul National University Seoul 151-921, Korea; 2 National Institute of Crop Science, Rural Development Administration Suwon, 441-857, Korea; 3 Department of Plant Pathology, Pennsylvania State University, University Park PA 16802, USA

## Abstract

*A**grobacterium tumefaciens-*mediated transformation (ATMT) has become a prevalent tool for functional genomics of fungi, but our understanding of T-DNA integration into the fungal genome remains limited relative to that in plants. Using a model plant-pathogenic fungus, *Magnaporthe oryzae*, here we report the most comprehensive analysis of T-DNA integration events in fungi and the development of an informatics infrastructure, termed a T-DNA analysis platform (TAP). We identified a total of 1110 T-DNA-tagged locations (TTLs) and processed the resulting data via TAP. Analysis of the TTLs showed that T-DNA integration was biased among chromosomes and preferred the promoter region of genes. In addition, irregular patterns of T-DNA integration, such as chromosomal rearrangement and readthrough of plasmid vectors, were also observed, showing that T-DNA integration patterns into the fungal genome are as diverse as those of their plant counterparts. However, overall the observed junction structures between T-DNA borders and flanking genomic DNA sequences revealed that T-DNA integration into the fungal genome was more canonical than those observed in plants. Our results support the potential of ATMT as a tool for functional genomics of fungi and show that the TAP is an effective informatics platform for handling data from large-scale insertional mutagenesis.

## Introduction

Since the completion of genome sequencing of the budding yeast *Saccharomyces cerevisiae* (Goffeau *et al*., 1996), over 50 fungal genome sequences have been released, with more than 20 additional sequencing projects currently underway (Park *et al*., 2006). This wealth of genome sequence data accelerates the progress of studying fungal biology via genome-wide mutagenesis of fungal genes in both random and targeted manners. Although the most efficient way of studying the function of individual genes is to disrupt the gene of interest and detect any resulting phenotypic alterations, genome-wide targeted mutagenesis has been reported only for *S. cerevisiae* (Winzeler *et al*., 1999; Giaever *et al*., 2002), mainly due to its idiosyncratically high efficacy for homologous recombination (Baudin *et al*., 1993).

An alternative approach is a large-scale, random insertional mutagenesis using restriction enzyme-mediated integration (REMI) or transposon-based methodologies (Sanchez *et al*., 1998; Sweigard *et al*., 1998; Hamer *et al*., 2001; Villalba *et al*., 2001). However, these mutagenesis techniques have limitations that render them inappropriate for large-scale functional genomic analysis, which include unsolicited deletion and rearrangement of DNA (Mullins *et al*., 2001) and biased insertion patterns (Hamer *et al*., 2001). Recently, *Agrobacterium tumefaciens*-mediated transformation (ATMT) has been widely used as a means of large-scale insertional mutagenesis of fungi (Idnurm *et al*., 2004; Walton *et al*., 2005; Blaise *et al*., 2007). *A. tumefaciens* is a plant-pathogenic bacterium that is capable of transferring part of its plasmid, a region known as the T-DNA, into the genome of host plants. ATMT has been used to produce a large number of insertional mutant plants in *Arabidopsis* (Alonso *et al*., 2003) and rice (Jeon *et al*., 2000; Sallaud *et al*., 2004), demonstrating its utility as a functional genomics tool. The host range of *A. tumefaciens* can be extended to include *S. cerevisiae* (Bundock *et al*., 1995) and diverse filamentous fungi (de Groot *et al*., 1998). With its versatility in transforming various fungal tissues and high efficiency, the number of successful fungal transformations via ATMT has rapidly increased (up to 64 species) in the past 5 years (Michielse *et al*., 2005; Lacroix *et al*., 2006).

The primary goal of large-scale random insertional mutagenesis is to generate mutations in most genes in a genome for functional studies. To evaluate the potential of T-DNA as an insertional mutagen in plants and to understand the mechanisms and characteristics of T-DNA integration, flanking sequences of many T-DNA insertion sites have been characterized (Alonso *et al*., 2003; Schneeberger *et al*., 2005; Zhang *et al*., 2007). In plants, T-DNA integration exhibited a strong bias towards the 5′ upstream regions of genes (Alonso *et al*., 2003; Schneeberger *et al*., 2005; Zhang *et al*., 2007). Analysis of junction sequences showed that the right border (RB) of T-DNA tends to be more conserved than the left border (LB), presumably resulting from the attachment of VirD2 proteins to RB, and that filler sequences and microhomology exist (Forsbach *et al*., 2003; Kim *et al*., 2003). In addition, tandem arrays of multiple T-DNA insertions, deletion of T-DNA target sites, translocation associated with T-DNA insertion, and simultaneous integration of T-DNA with the vector backbone have also been observed (Forsbach *et al*., 2003; Kim *et al*., 2003).

In fungi, information on the pattern of T-DNA integration has been very limited, with only a few T-DNA insertion sites having been characterized in several species transformed using *A. tumefaciens* (Bundock *et al*., 1995; Mullins *et al*., 2001; Leclerque *et al*., 2004). Analysis of ∼100 T-DNA transformants of *Leptosphaeria maculans* was conducted to elucidate T-DNA integration patterns in this filamentous fungus (Blaise *et al*., 2007). Although this analysis included the largest number of transformants characterized to date, its scope was still limited and insufficient for a comprehensive understanding of T-DNA integration patterns in filamentous fungi. Here we describe the analysis of T-DNA insertion sites in 1246 transformants of *Magnaporthe oryzae*, and the parallel development of a web-accessible informatics system, termed the T-DNA analysis platform (TAP), that serves as an electronic warehouse and an analysis pipeline for T-DNA integration data. *M. oryzae* causes rice blast disease and is an important model organism for investigating fungal infection-related development and pathogenicity because of its genetic tractability (Talbot, 2003). The genomic sequences of both the fungus (Dean *et al*., 2005) and rice (Yu *et al*., 2002) are available, providing a unique opportunity to study a host–parasite interaction from both sides using functional genomics approaches.

Handling and sharing large volumes of T-DNA insertion data in plants require the development of an informatics platform supporting data management and analysis. Several databases like FLAGdb, ATIDB, RiceGE and GABI-KAT have been developed to address such needs (An *et al*., 2003; Pan *et al*., 2003; Samson *et al*., 2004; Li *et al*., 2007). However, to our knowledge, a comparable informatics platform for fungi has not been reported. Our systematic analysis of the distribution and patterns of T-DNA integration in the *M. oryzae* genome through combination of biological data with the TAP will serve as a model system for ATMT-mediated functional genomic analysis of fungi.

## Results

### Construction of the TAP and characterization of T-DNA-tagged locations

We recently reported the generation of 21 070 transgenic *M. oryzae* strains using ATMT and provided a comprehensive phenotypic analysis of these transformants (Jeon *et al*., 2007). To gain insight into T-DNA integration patterns in *M. oryzae*, we analysed chromosomal location and structure of inserted T-DNA in 1246 transformants, including 174 randomly selected transformants (RSTs) and 1109 transformants exhibiting phenotypic defects transformants (PDTs) in the previous screening (35 of them were coincidentally shared between two groups). From these transformants, thermal asymmetry interlaced-PCR (TAIL-PCR) produced 2116 readable sequences, of which 1439 (68.0%) contained both a T-DNA border and fungal genome DNA, and 587 (27.7%) and 90 (4.3%) sequences only contained genomic DNA and T-DNA/vector sequences respectively (Table S1). Thus, over 95% (2026 of 2116) of sequences were suitable for determining T-DNA-tagged locations (TTLs) in the *M. oryzae* genome. The sequences without border were still regarded as genomic sequences existing adjacent to T-DNA's border, because they were rescued by border-specific primers. The remaining 90 sequences with no matches to *M. oryzae* genome sequences were excluded from subsequent analyses (Table S1).

**Fig. 1:**
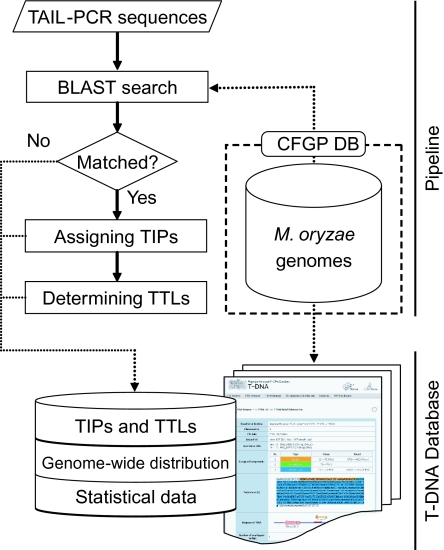
Organization of the TAP and the flow chart of sequence data processing. The TAP consists of a data analysis pipeline, a T-DNA database and a user interface. The automated process of analysing rescued sequences (sequences of TAIL-PCR products) via the pipeline is shown in a flow chart. Those sequences are matched to a blast search, and then the parser program extracts the following information: chromosome number, type of border, integrated positions and TTLs. The T-DNA database transforms the insertion information to reveal genome-wide distribution patterns. Outputs are shown via a user-friendly web interface. Solid arrows indicate the processes of the pipeline, and dotted arrows denote data flow.

To effectively archive and analyse data from these TTLs, we developed an informatics platform, termed the TAP, consisting of a data analysis pipeline, a T-DNA database and a user-friendly web interface. The TAP was designed to effectively use the *M. oryzae* genome sequence data stored in the in-house genome data warehouse, called the comparative fungal genomics platform (CFGP; http://cfgp.snu.ac.kr; J. Park *et al*., unpublished) (Fig. 1), for analysing patterns and genome features associated with T-DNA insertion. The analysis pipeline conducted a two-step analysis for individual sequences rescued from T-DNA insertion sites. In the first step, junction structure and chromosomal position of each T-DNA insertion were determined through blast searches against the *M. oryzae* genome sequence data and the pBHt2 vector, a binary vector used in ATMT. In the second process, any sequences shorter than 26 bp were filtered out (Sessions *et al*., 2002), and multiple insertion positions within a 35 bp window were clustered as one TTL to prevent overestimation of the number of independent TTLs.

The T-DNA database was designed to store information associated with individual TTLs and to provide a user-friendly interface allowing visualization of T-DNA insertion patterns and flanking sequences for each TTL. The GC ratio, CG/AT skew and DNA bendability (see below) around each insertion site were made available for viewing. In addition, statistical data, including chromosomal distribution of TTLs and TTL frequency, were also included in the TAP to illustrate overall features associated with T-DNA insertion in the *M. oryzae* genome. The data described below were generated in real time using the TAP.

### Identification of TTLs in the *M. oryzae* genome

**Fig. 2:**
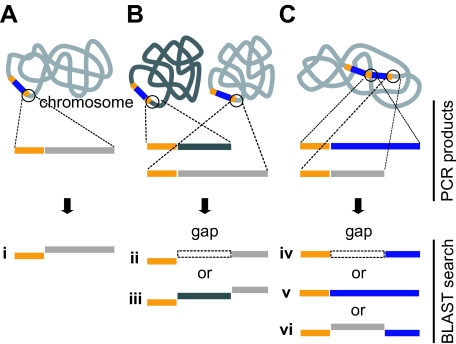
Identification schemes for T-DNA junction types in *M. oryzae*. TAIL-PCR products from transformants with a single T-DNA insertion (A), multiple insertions at different locations (B), or multiple tandem or inverted insertions (C) were sequenced. Resulting sequences were subjected to blast searches (black vertical arrow) and analysed for junction type (see *Experimental procedures*). Those that produced a single fragment in TAIL-PCR and matched a T-DNA border (yellow) and a flanking genomic region (grey) without a gap were considered ‘precise junctions’ (type i) (51). In some cases, multiple fragments were amplified in TAIL-PCR at two different genomic regions (type iii; grey and dark grey). The transformant was regarded as having mixture of ‘precise’ and ‘imprecise’ junctions (types i and ii respectively), and treated as two TTLs. The imprecise junction was defined as the sequence matched to both a border and a flank with a gap (type ii) (51). When multiple T-DNA (blue bar with yellow borders) were integrated into the same location with tandem or inverted arrays, multiple flanking regions could appear (C). According to the concentration of co-amplified PCR products, sequencing results were classified as type ii (similar in both dark and light grey products), type iii (more dark grey product), type iv (similar in both grey and blue product), type v (more blue product) or type vi (more grey products). In the blast search, different locations or kinds of matching results were shown by the different line levels and colours.

Whereas 70 flanking sequences (3.5% of 2026) matched with repetitive sequences such as MsR02/RETRO7, MAGGY and MGLR3/MG-SINE transposons (see *Experimental procedures*), the majority (96.5%) of TTLs corresponded to single-copy regions of the genome. Although in most transformants, a single copy of T-DNA was inserted at one locus (82% in Table S2; Fig. 2A), integrations of T-DNA at multiple loci and an insertion of tandem-repeated T-DNAs in one locus were also observed (Fig. 2B and C respectively, and Table S2).

For determination of TTLs, we adopted the definitions used for analysing T-DNA integration patterns in *Arabidopsis* (Mayerhofer *et al*., 1991), in which each junction between the T-DNA border and genomic DNA was classified as ‘precise’ or ‘imprecise’. In defining ‘imprecise’ junctions, we included junctions containing a stretch of undeterminable base calls or another genomic region (a gap in Fig. 2B and C), whereas Mayerhofer *et al*. (1991) included only filler sequences of unknown origin. Junctions exhibiting a T-DNA border immediately joined to a single region of the *M. oryzae* genomic DNA were designated as ‘precise’, and their TTLs were assigned at the beginning of flanking genomic sequences. In the case of ‘imprecise’ junctions, corresponding TTLs were determined as the point next to the border end. Of 1439 sequences, 845 and 594 were determined to be precise and imprecise junctions respectively; in total, they corresponded to 764 independent TTLs (Table S1). Sequences composed only of *M. oryzae* genomic DNA sequences were also included in TTL determination, with the starting position of a sequence being defined as the insertion site. The 587 genomic DNA-only sequences led to 346 independent TTLs (Table S1). A total of 1110 TTLs were identified from the 2026 sequences derived from 1246 transformants.

### Genome-wide features associated with TTL distribution

We analysed the distributions of 1110 TTLs on chromosomes, and in genic and intergenic regions, based on the *M. oryzae* genome in the CFGP. Because most of the TTLs were derived from phenotype-defective transformants, we first tested overall randomness in the RST and PDT groups. The distribution patterns of TTL frequency between two groups displayed a clear correlation (*r* = 0.303, *P* < *0.05* in Pearson method), but neither correlated with a purely random model generated through Monte Carlo simulation (data not shown). In addition, two groups exhibited almost identical distribution patterns on chromosomes and in genetic elements (Tables S3 and S4). Thus, all TTLs were pooled and subsequently analysed as one group.

**Fig. 3:**
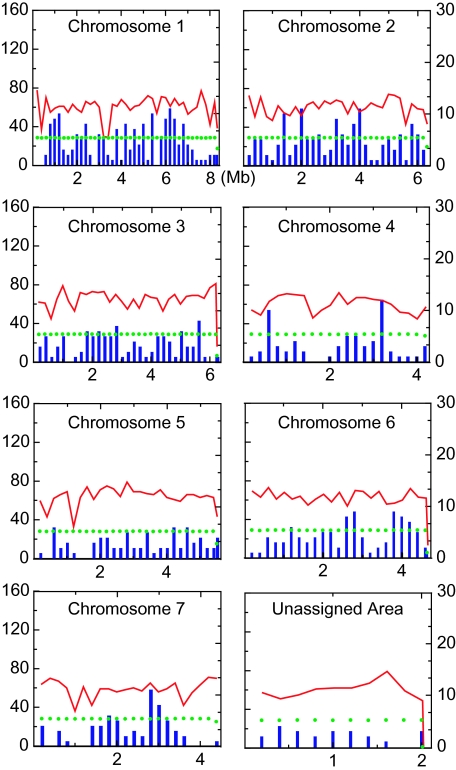
Distribution of 1110 TTLs across *M. oryzae* chromosomes. The frequency of TTLs (blue bar) and expected (based on the random insertion) T-DNA insertions (green dot) in every 200 kb are plotted along the length of each chromosome. Gene density on each chromosome is indicated as a red line. Frequencies of expected insertions were estimated by Monte Carlo simulations. The unassigned area includes the TTLs that matched the 2.01 Mb of unmapped genome sequences. The length of each chromosome is indicated on the *x*-axis, and frequencies of genes and TTLs are indicated on the left and right *y*-axes respectively.

To confirm whether T-DNAs were evenly distributed, 10 000 simulations using Monte Carlo methods were performed based on a purely random model (Fig. 3). The distribution of simulated samples (green dots) showed no significant correlation to that of observed TTLs (blue bars), indicating that the TTL distribution in this organism did not follow the purely random model (*r* = 0.154, *P* < *0.05*). When analysed at the chromosomal level, 44% of the total TTLs were observed on chromosomes 1 and 2 (one in every 30 kb), which was higher compared with other chromosomes, and exceeded the expected numbers by more than 20% (Table 1). In contrast, chromosomes 4, 5 and 7 contained less than 80% of the expected T-DNA insertions (one insertion in every 50 kb; Table 1). These tendencies were significant in chi-squared tests as indicated by the resulting *P*-values (Table 1). In addition, TTL frequency had no specific association with the following characters of the genome: gene density (red lines in Fig. 3), GC ratio, transposable elements and microsatellites (data not shown; see *Experimental procedures*).

**Table 1:** Chromosomal distribution of T-DNA-tagged locations.

Chromosome	Length (Mb)	No. of observed TTLs	No. of expected TTLs^a^	Insertion interval (kb per insertion)	Value of χ^2^^b^	*P*-value
1	8.32	276	222	30.1	13.2	0.00*
2	6.33	207	169	30.6	8.6	0.00*
3	6.24	164	166	38.0	0.0	0.85
4	4.19	89	112	47.1	4.6	0.03*
5	5.51	116	147	47.5	6.5	0.01*
6	4.64	142	124	32.7	2.7	0.10
7	4.38	86	117	50.9	8.1	0.00*
Unassigned area	2.01	30	53	67.0	10.0	0.00*
Total	41.62	1110	1110	37.5	–	–

a Expected numbers were calculated according to the chromosome length.

b Chi-squared test is based on the difference between observed and expected values. Low *P*-values mean that they are significantly different from the expectation. Chi-squared values are calculated as following function: χ^2^ = Σ{(observed TTLs – expected TTLs)^2^/expected TTLs}. *P*-values are calculated with degree of freedom, 1.

* Significant at *P* < 0.05.

**Fig. 4:**
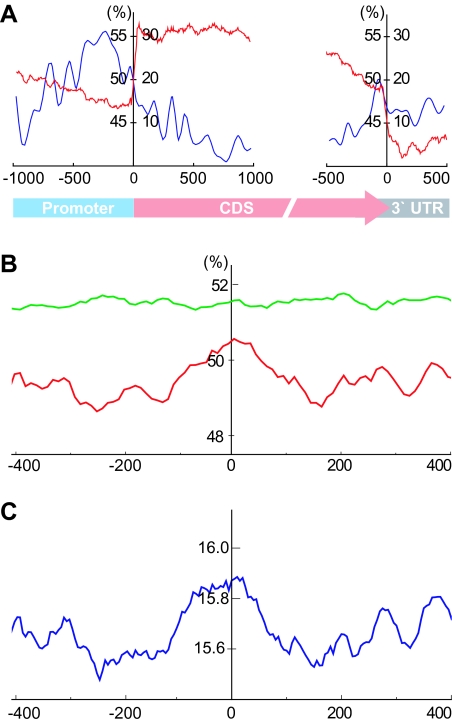
Characteristics of T-DNA insertion sites. A. Distribution of TTL frequency and GC ratio in the genic region (blue and red lines respectively). The profile of TTL frequency was summed for every 50 bp unit, and that of the GC ratio was calculated for 50 bp windows at 5 bp intervals. The position 0 on the *x*-axis indicates the CDS start and end points. Negative numbers indicate upstream regions of the CDS start or end, whereas positive numbers indicate areas downstream from those points. On the *y*-axis, the left side presents the GC ratio and the right side indicates the TTL frequency. B. GC ratio profile around T-DNA insertion sites. The GC ratio was analysed for the 800 bp region flanking T-DNA insertion sites. Centred on the T-DNA insertion site, the profile is plotted as a red line. It was compared with the control profile of GC ratio generated from randomly selected locations (green line). Each point shows an average GC ratio of the 50 bp window around the point. The position 0 indicates the T-DNA insertion site. Negative and positive numbers on the *x*-axis indicate upstream and downstream of T-DNA insertion sites respectively. C. The bendability profile around T-DNA insertion sites. Bendability of the 800 bp region flanking T-DNA insertion sites was analysed. The window moved at 5 bp intervals through this region. High bendability indicates a more flexible region for T-DNA integration. The scale of the *x*-axis is the same as that of the axis in B.

We analysed the distribution of TTLs in the genic and intergenic regions. More TTLs were observed in the genic region than in the intergenic region of the *M. oryzae* genome (799 and 311 respectively; Table 2). As supported by the chi-squared test (Table 2), the observed numbers represented 94% and 120% of the expected numbers, suggesting that T-DNA integration seemed to be slightly biased towards the intergenic region in *M. oryzae*. Within the genic region, a higher frequency of T-DNA insertions was observed in the promoter region (defined as 1 kb upstream of the transcriptional starting point; 415 of 799) than the coding or 3′ untranslated regions (UTRs, defined as 500 bp downstream of end codon; 256 and 128 respectively; Table 2). The observed number of TTLs in the promoter region was twofold higher than the expected number of TTLs, suggesting a strong bias towards T-DNA insertions in this region. Among the 256 TTLs in coding sequences (CDS), 196 (77%) and 60 (23%) were found in the exon and intron regions respectively; the observed frequencies were negatively correlated with the expected value (43% and 76% respectively). To further investigate the nature of bias in the genic region, TTL frequencies around the start and the end of CDS were examined (blue line in Fig. 4A). At the start of CDS, more TTLs were found in the promoter region (average 25.7) than in the CDS region (average 11.3). A similar tendency of TTL distribution was found around the end of CDS (12.7 in 3′ UTR and 10.4 in CDS on average of 500 bp region). To determine whether this TTL pattern exhibited any relationship with base composition, the GC ratio was analysed. The GC ratio (red line in Fig. 4A) was plotted over the TTL frequencies, which showed dramatic changes at the start and end of CDS. Near the CDS start, the GC ratio increased sharply from 47% to 56% within 100 bp, and the number of TTLs decreased from 23 to 15. Near the CDS end, the overall tendency was also negatively correlated (Fig. 4A). In summary, a high GC ratio appeared to be negatively correlated with T-DNA integration, suggesting that T-DNA preferred AT-rich regions.

**Table 2:** Distribution of T-DNA-tagged locations around genes.

Type of region	Length (kb)	No. of observed TTLs	No. of expected TTLs^a^	Density (no. per Mb)	Value of χ^2^^b^	*P*-value
Genic	31 954	799	852	25.0	3.31	0.07
Coding region	19 888	256	530	12.9	141.93	0.00*
Exon	16 937	196	451	11.6	144.72	0.00*
Intron	2 951	60	79	20.3	4.44	0.04*
5′ promoter (< 1 kb)	6 613	415	176	62.8	322.95	0.00*
3′ UTR (< 0.5 kb)	5 453	128	146	23.5	2.05	0.15
Intergenic	9 667	311	258	32.2	10.98	0.00*
Total	41 624	1110	1110	26.7		

a Expected numbers were calculated according to the chromosome length.

b Chi-squared test is based on the difference between observed and expected values. Low *P*-values mean that they are significantly different from the expectation. Chi-squared values are calculated as following function: χ^2^ = Σ{(observed TTLs – expected TTLs)^2^/expected TTLs}. *P*-values are calculated with degree of freedom, 1.

* Significant at *P* < 0.05.

### Characteristics of the *M. oryzae* genome around TTLs

To examine characteristics near TTLs, the 0.8 kb sequences flanking individual TTLs were integrated into a dataset. This dataset was compared with a control set that consisted of the same amount of sequence data from random positions which have been generated using the Monte Carlo method with 10 repeats. The GC ratios were first profiled for these two datasets using a 50 bp window with 5 bp intervals (Fig. 4B). Overall, the GC ratio (red line) was lower than that of the control dataset (average 51.5%, green line), again supporting the positive correlation between T-DNA insertion and AT-rich regions. Another feature was that a relatively high GC ratio was observed in the vicinity of TTLs (± 50 bp from the insertion position), compared with the remaining areas (Fig. 4B). This suggested that the vicinity of T-DNA insertion sites differed from the other regions in terms of the base composition. Second, the DNA bending property, or bendability, was analysed for these sequence datasets, because recent reports suggested a relationship between bendability and T-DNA insertions (Schneeberger *et al*., 2005; Zhang *et al*., 2007). Two different analyses to predict DNA bendability (Satchwell *et al*., 1986; Brukner *et al*., 1995a) were performed, and similar bendability profiles appeared in both analyses. One of them is shown in Fig. 4C. Regions proximal to TTLs (average bendability = 15.9) were more bendable than distant regions (15.6, where smaller values indicate less bending). These results suggested that T-DNA might favour more flexible or bendable regions for insertion.

### Analysis of junction structures

To characterize junction structures formed by T-DNA integration, 845 precise junctions (246 LB and 599 RB) were analysed. A total of 493 sequences (161 LB and 332 RB) were determined to represent independent insertion positions and were used to analyse the nature of T-DNA border truncation, microhomology between T-DNA and host genomic DNA, and genomic DNA deletion.

**Fig. 5:**
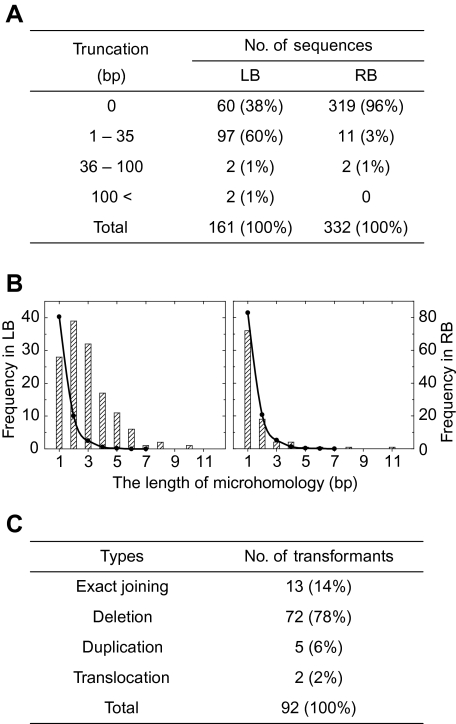
Border truncation, microhomology and host genome deletion at 493 precise junctions. A. Frequency distribution of T-DNA border truncations. B. Distribution of the length of nucleotides identical between genomic and border sequences (microhomology). The expected length of microhomology was plotted on the basis of a random simulation (thick lines). C. Frequency distribution of host genome deletions at 92 insertion sites where sequences at both sides of insertion were determined.

Left and right T-DNA borders from the selected 493 precise junctions included truncation from the known VirD2 cleavage sites. The LB region was truncated at a high frequency (62%, 101 of 161; Fig. 5A), with the length of deletions ranging from 1 to 147 bp. On the other hand, the RB region appeared well preserved (4% 13 of 332; Fig. 5A), with the length of deletions ranging from 1 to 74 bp. Small truncations with less than 35 bp represented 97.0% and 84.6% of the LB and RB truncations respectively, with only a few truncations exceeding 35 bp at both ends of the T-DNA (Fig. 5A).

T-DNA insertion points in the *M. oryzae* genome were accurately determined by blast search. Comparisons between T-DNA end sequences and sequences at insertion sites revealed that T-DNA integration was often associated with microhomology between genomic DNA and either end of the T-DNA (Fig. 5B). Microhomology was observed more frequently in the LB region (85%, 131 of 161) than in the RB region (31%, 102 of 332). Microhomology reached up to 11 bp, but was usually less than 5 bp (Fig. 5B), indicating that T-DNA integration in *M. oryzae* does not require long stretches of homology at the cross-over point as implicated in illegitimate recombination in yeast, plants and mammals (Mayerhofer *et al*., 1991; Bundock *et al*., 1995; Kunik *et al*., 2001).

Analysis of the 92 insertion sites from which sequences of both sides of the integrated T-DNA were obtained showed that exact joining (without deletion, addition and microhomology) of T-DNA and genomic DNA was observed in 13 (14.1%) integration events (Fig. 5C). Deletion of genomic DNA at the insertion site ranged from 1 to 1950 bp and occurred frequently (78.3%, 72 of 92; Fig. 5C). Most deletions (77.8%, 56 of 72) were less than 35 bp; those larger than 100 bp comprised only 9.8% (9 of 72; Fig. 5C). In addition, small duplications (1–11 bp) and chromosomal translocations were also observed at five and two insertion sites respectively (Fig. 5C).

### T-DNA integration patterns in the *M. oryzae* genome

**Fig. 6:**
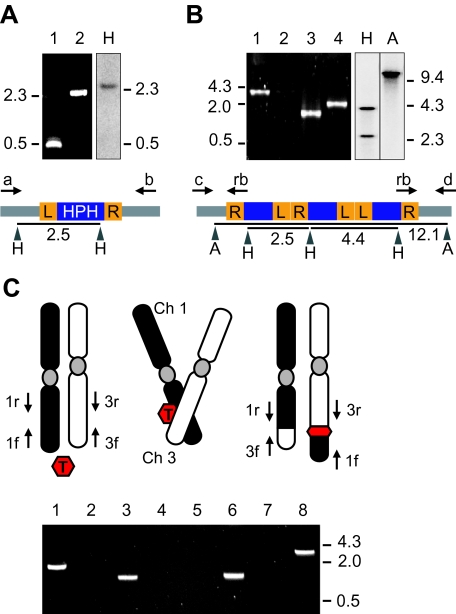
Patterns of T-DNA integration. A. A single T-DNA integration. T-DNA is shown as a blue bar enclosed with yellow borders. The expected restriction enzyme sites are indicated by triangles. Southern blot analysis of transformant ATMT0879C5 (lane H) revealed a single T-DNA integration, resulting in a ∼2.3 kb hybridizing band. A pair of flanking primers (arrow; a and b) amplified a 2.5 kb band in the transformant (lane 2), compared with wild-type (lane 1). B. Multiple T-DNA integration at one site. Southern blot analysis confirmed the insertion of three T-DNAs in one location (ATMT0060C3), based on two restriction enzymes that cut inside (lane H) or outside (lane A) the T-DNA. A pair of flanking primers (c and d) amplified a 4.0 kb band in wild-type (lane 1) but no band in the transformant (lane 2). Additionally, the combination of border and flanking primers (c-rb and d-rb) proved that both sides of the repeated T-DNAs ended with RB (lanes 3 and 4). C. Chromosomal rearrangement. The translocation between chromosomes 1 and 3 occurred in transformant ATMT0879C6 as a result of T-DNA integration (red hexagon). This rearrangement was confirmed by PCR amplification using mixed combination of deduced primers specific for flanking areas (lanes 1 and 2, 1f/1r; lanes 3 and 4, 3f/3r; lanes 5 and 6, 1f/3r; lanes 7 and 8, 3f/1r). Wild-type DNA (lanes 1, 3, 5 and 7) was compare with the transformant (lanes 2, 4, 6 and 8). In all figures, H and A indicate *Hin*dIII and *Apa*I respectively. The unit of number of markers and length indicator is kilobase.

Several abnormal patterns of T-DNA integration were observed. In 70 transformants with a known T-DNA copy number and sequences flanking both sides of the T-DNA, 57 (81%) had single T-DNA integration (Fig. 6A) and the remaining 13 harboured more than two copies of T-DNA. Abnormal patterns were found in 9 out of 70 (13%) transformants, where four were confirmed to have direct or inverted repeats of T-DNA, and five single-copy transformants exhibited translocation, fragment insertion or readthrough patterns.

Transformant ATMT0060C3 is an example showing multiple T-DNA integration in both tandem and inverted manners. Southern blot analysis produced two and one band(s) when genomic DNA digested with the restriction enzymes *Hin*dIII and *Apa*I respectively (Fig. 6B, lanes H and A). The tandem-repeated T-DNAs yielded a ∼2.5 kb band, and the inverted repeat (LB to LB) gave a ∼4.4 kb band (lane H). Both sides were identified by PCR methods with RB-specific primer (rb) and deduced flanking-specific primers (c and d). A primer combination of rb-c and rb-d (lanes 3 and 4) confirmed the T-DNA insertion, whereas the corresponding band appeared in the wild-type, not in the transformant because the repeated T-DNA was too large to be amplified (lane 1 and 2). Readthrough samples were found in two transformants. Transformant ATMT0144A2 had a binary vector backbone sequence adjacent to the RB cleavage site accompanied by repeated T-DNAs. LB readthrough was also detected in one transformant that had a single copy of T-DNA (ATMT0156D5).

Two in 70 transformants (3%) had translocations as a result of T-DNA integration. Both cases involved two different chromosomes, resulting in chromosome rearrangements. Transformant ATMT0879C6 had LB and RB flanking sequences that matched sequences in chromosomes 1 and 3 respectively (Fig. 6C). The primer sets (1f/1r and 3f/3r) amplified bands from the wild-type strain (Fig. 6C, lanes 1–4), which were absent in the transformant, whereas the exchanged sets (1f/3r and 3f/1r) amplified bands only in the transformant (Fig. 6C, lanes 5–8).

## Discussion

In combination with rapidly increasing fungal genome sequence data, ATMT has been proposed as a powerful tool for fungal functional genomics (Michielse *et al*., 2005; Lacroix *et al*., 2006). The recent application of ATMT in a large-scale mutant hunt in *M. oryzae* supported this proposition (Jeon *et al*., 2007). Despite its potential, only limited information is currently available on the patterns and mechanisms of T-DNA integration in fungi (Bundock *et al*., 1995; Bundock *et al*., 2002). A comprehensive informatics platform for archiving and analysing the anticipated flood of T-DNA-tagged sequences is needed to maximize the utility of ATMT (Walton *et al*., 2005; Blaise *et al*., 2007). In this study, using *M. oryzae* as a model fungal host for ATMT, we comprehensively analysed 2116 sequences associated with T-DNA insertion sites via a novel informatics platform called the TAP, which can also be utilized to handle T-DNA flanking sequences from other organisms.

TAIL-PCR was used to rescue genomic sequences flanking T-DNA insertion sites because of its convenience in high-throughput processing of a large number of transformants (Singer and Burke, 2003). Among 2116 readable sequences, 2026 (96%) matched genomic regions of *M. oryzae* (Table S1). The success rate in isolating T-DNA flanks from *M. oryzae* was higher than those reported in plants, which ranged from 60% to 80% in large-scale experiments (Sallaud *et al*., 2004). Presumably, this was a result of the relatively simple T-DNA integration pattern in this fungus compared with plants.

Although T-DNA integration was initially thought to be random, its randomness has been controversial with recent data indicating non-randomness of T-DNA insertion (Tinland, 1996; Michielse *et al*., 2005). Early studies based on small number of samples indicated random distributions of T-DNA insertions throughout target genomes (Thomas *et al*., 1994; AzpirozLeehan and Feldmann, 1997; Barakat *et al*., 2000; Bundock *et al*., 2002). However, more recent large-scale studies based on over 1000 sequences have indicated that T-DNA insertions are not truly random relative to genetic elements and sequence compositions (Sessions *et al*., 2002; Chen *et al*., 2003). Our genome-wide analysis using 1110 TTLs also indicated certain biases in both genic distribution and sequence composition. T-DNA insertions were biased towards promoters and against gene-coding regions. In addition, overall GC ratios around the insertion sites were lower than simulated control values. Such T-DNA preference was thought to be related to the base composition of insertion sites, because eukaryotic promoters and gene-coding regions are relatively well conserved as AT and GC-rich regions respectively (Hurst *et al*., 2004). In rice and *Arabidopsis*, T-DNA insertions were favoured in promoter, 3′ UTRs and intergenic regions, all of which are relatively AT-rich (Alonso *et al*., 2003; Sallaud *et al*., 2004; Pan *et al*., 2005). Thus, this distinct bias towards promoters or AT-rich regions may not be specific to certain organisms, but may be a conserved feature in the T-DNA integration mechanism.

As noted in previous studies of *Arabidopsis* and rice (Schneeberger *et al*., 2005; Zhang *et al*., 2007), the prominent peak in predicted bendability around T-DNA insertion sites in our study supports the reasoning that regions with higher flexibility are preferred targets for T-DNA integration. As a physical property, bendability plays important roles in interactions between DNA sequences and DNA binding proteins, such as nucleosome and DNase I (Satchwell *et al*., 1986; Brukner *et al*., 1995a). Previous reports suggested that highly bendable regions promote open chromatin structure and active transcription (Widlund *et al*., 1999; Vinogradov, 2003). Although it is unclear whether T-DNA integrates directly to open chromatin, findings to date suggest that DNA bendability is one of the factors contributing to T-DNA preference for the promoter regions, where RNA polymerase has been known to interact actively (Dlakic *et al*., 2005).

In a few cases, as consequences of T-DNA integration, various genetic abnormalities, such as tandem or inverted repeats of T-DNA, readthroughs and chromosomal rearrangements, were observed in the genome of *M. oryzae*. Prevalent occurrence of truncation and microhomology at LBs and frequent deletions of host genome sequences around insertion sites were also consistent with findings in plants (Forsbach *et al*., 2003; Kim *et al*., 2003). However, overall frequencies and extents of such genetic changes were lower in *M. oryzae* than in plants. For example, intactness of over 96% of the inserted RBs following integration was in great contrast to what was observed in *Arabidopsis* and rice (19–57% intactness) (Brunaud *et al*., 2002; Forsbach *et al*., 2003; Kim *et al*., 2003). Compared with *Arabidopsis*, the majority of deletions of host DNA in *M. oryzae* occurred at much smaller scales (11–100 bp versus 1–35 bp), and the rate of exact joining between the T-DNA border and host DNA was five times higher (14% versus 2.7%). The well-conserved RBs and the highly asymmetric frequency of microhomology between borders in *M. oryzae* support the current hypothesis on the mechanism of T-DNA integration, in which the VirD2 protein protects the RB from degradation by binding to this end of T-DNA, and integration occurs through illegitimate (non-homologous) recombination (Mayerhofer *et al*., 1991; Tinland, 1996). Our results suggest that *M. oryzae* is an excellent model for studying the molecular mechanisms underpinning T-DNA integration.

The generation of a tremendous number of sequences from T-DNA-tagged sites in plants necessitates the construction of databases to store resulting data (An *et al*., 2003; Pan *et al*., 2003; Samson *et al*., 2004; Li *et al*., 2007). The anticipated increase in T-DNA-tagged sequences from fungi now demands a similar informatics platform. The TAP serves as a depository for the enormous number of sequences generated in this study. In addition, it carries out automatic analyses of deposited sequences to provide multiple contexts associated with T-DNA insertion sites, such as genome-wide distribution of TTLs with density views, base composition and bendability around insertion sites. The data analysis functions provided by the TAP make it unique compared with previous ATMT databases for plants. Furthermore, the TAP was designed to store and handle multiple datasets from any genome sequence; thus, it is capable of providing comparative insight into T-DNA integration for any organism.

Overall, our data suggest that non-random and promoter-favourable T-DNA integration can be significantly affected by AT-rich base composition and DNA bending properties. Simpler integration patterns and structures make this fungus a better model to understand the mechanisms of T-DNA integration. Our informatics platform can be extended to other fungal systems, allowing comprehensive insight into T-DNA integration mechanisms in fungi and plants.

## Experimental procedures

### Fungal transformants and media

T-DNA-tagged transformants generated via ATMT were obtained from the Center for Fungal Genetic Resources (Seoul National University, Korea; http://genebank.snu.ac.kr). Transformants were generated from *M. oryzae* strain KJ201 using *A. tumefaciens* strain AGL-1 harbouring the pBHt2 vector (Mullins *et al*., 2001; Rho *et al*., 2001; Jeon *et al*., 2007). All transformants were stored dry as previously described (Jeon *et al*., 2007). Fungal cultures, activated using V8 juice agar, were grown in liquid complete media for genomic DNA isolation.

### Large-scale genomic DNA extraction

Genomic DNA extraction was performed as previously described (Rogers and Bendich, 1985) using a 24-prong plastic grinder customized to fit 24-well plates (Falcon Multiwell; Becton Dickinson, Franklin Lakes, NJ) (Jeon *et al*., 2007). Restriction enzyme digestion, agarose gel electrophoresis and DNA gel blotting were performed following standard procedures (Sambrook and Russel, 2001). Hybridization was carried out for individual isolates using the hygromycin B phosphotransferase (*hph*) gene cassette as a probe following the previously described procedures (Kim *et al*., 2005).

### TAIL-PCR and sequencing

TAIL-PCR was used to isolate the T-DNA flanking sequence with some modification of both the original (Liu and Whittier, 1995) and modified TAIL-PCR protocols (Sessions *et al*., 2002; Singer and Burke, 2003). T-DNA border-specific primers and arbitrary degenerate (AD) primers are described in Table S5. The final concentration of specific primers was adjusted to 0.2 and 0.4 μM in primary and secondary (or tertiary) reactions respectively, and that of AD was 3–4 μM depending on its degeneracy. For high-throughput amplification, TAIL-PCR was performed for individual isolates in the 96-well plate, and AD primer pools, such as ADM1 (AD1 : AD2 : AD3 = 4:3:3) or ADM2 (AD1 : AD4 : AD6 = 3:4:4), were used to increase the efficiency of rescuing sequences flanking inserted T-DNAs. T-DNA flanks were amplified with 1 unit of Taq DNA polymerase using 10–30 ng of genomic DNA as a template, and purified using ExoSAP-IT® (USB, Cleveland, OH, USA) according to the manufacturer's recommendation. The PCR products were sequenced using BigDye (Applied Biosystems, Foster City, CA) primer sequencing chemistry following the manufacturer's specifications and analysed on an ABI 377 DNA Sequencer (Applied Biosystems, Foster City, CA). Border-specific primers (LB3 and RB3), which were 71 and 82 bp from the known cleavage sites respectively, were used for sequencing.

### Automatic data processing via the TAP to identify TTLs

For the determination of T-DNA insertion sites, the sequences rescued by TAIL-PCR were automatically processed through the data analysis pipeline of the TAP. These sequences were compared with the combined set consisting of the *M. oryzae* genome and the pBHt2 vector sequences, using the blast program (BLASTN with the default cut-off of 0.01), all of which were available in CFGP. The latest *M. oryzae* genome annotation (release 5; http://www.broad.mit.edu/annotation/genome/magnaporthe_grisea) was stored in the CFGP. After parsing blast search results, border (RB or LB) and junction (precise, imprecise or vector) types were defined using PERL scripts. If the sequence did not include the border sequences, the start position of the matched genomic sequence was considered the insertion position. In cases of sequences matching with ‘repetitive areas’, where the same sequence matched with multiple areas of the genome, the location exhibiting the highest *e*-value was considered the insertion site.

The second process of the pipeline was the assignment of TTLs. In this process, insertions in which the matched sequence was shorter than 26 bp (*e*-value < 3e^−8^) and located within 35 bp of other insertions were excluded. Cut-off length of 26 bp was determined by experimental confirmation of positive T-DNA insertions (Sessions *et al*., 2002). The window of 35 bp was chosen based on characterization of 67 samples having imprecise junctions (Fig. 2, type iii). Low-quality base calling or matches to other regions (vector or genomic sequence) contributed to this error. All insertion information was stored in the T-DNA database (designed using MySQL) and analysed.

### Transposable elements and simple sequence repeat (SSR) analysis

To define transposable elements in *M. oryzae*, the sequences of 24 different transposable elements were retrieved (Thon *et al*., 2004; Thon *et al*., 2006) and analysed based on a homology search of the *M. oryzae* genome sequence. SSR sequences were characterized based on the previous criteria (Temnykh *et al*., 2001). The SSR markers of di-, tri-, tetra-, penta- and hexa-nucleotide repeats were identified in the *M. oryzae* genome sequences.

### Bendability analysis

To analyse DNA bendability, two models based on nucleosome position preference and DNase I sensitivity were used (Satchwell *et al*., 1986; Brukner *et al*., 1995a). For the former model, the ‘banana’ program in EMBOSS (Rice *et al*., 2000) was used and stored the result in a database. DNase I sensitivity was calculated using the trinucleotide parameter of DNA bending (Brukner *et al*., 1995b).

### Statistics

Monte Carlo tests were conducted with 10 000 replicates with a purely random model, which generated random insertion positions on seven *M. oryzae* chromosomes. Three different ways to assess correlation were used: Pearson correlation coefficient, Kendall's tau and Spearman's rho (Best and Roberts, 1975). Chi-squared tests were performed with one degree of freedom (Thomas *et al*., 1994). Pair-wise *t*-tests, a correlation test and linear regression were executed via ‘t.test’, ‘corr.test’, and ‘lm’ functions in the *R* package respectively (Chambers and Hastie, 1992).

## Footnote

During the review and revision of this article, two independent studies reporting T-DNA integration patterns in *M. oryzae* have been published (Li, G.H., Zhou, Z.Z., Liu, G.F., Zheng, F.C., and He, C.Z. (2007). Characterization of T-DNA insertion patterns in the genome of rice blast fungus *M. oryzae*. *Curr Genet*. 51: 233–243, and Meng Y., Patel, G., Heist, M., Betts, M.F., Tucker S.L., and Galadima, N. *et al*. (2007) A systematic analysis of T-DNA insertion events in *M. oryzae*. *Fungal Genet Biol*. doi:10.1016/j.fgb. 2007.04.002). The patterns of T-DNA distribution and T-DNA insertion reported in these papers were similar to our results.

## Supplementary material

This material is available as part of the online article from: http://www.blackwell-synergy.com/doi/abs/10.1111/j.1365-2958.2007.05918.x (This link will take you to the article abstract).
