# Supplementary material for: Genome-wide analysis of T-DNA integration into the chromosomes of Magnaporthe oryzae
Source: Mol Microbiol. 2007 Oct;66(2):371–82. doi: 10.1111/j.1365-2958.2007.05918.x (PMC2169514; doi:10.1111/j.1365-2958.2007.05918.x)
Supplement: Supplementary file 1 [file mmi0066-0371-SD1.pdf]

Table S1. Summary of readable sequences from TAIL-PCR

Type of sequences	No. of sequences	No. of TTLs
Border + Genomic sequence	1,439 (68%)	764 (69%)
Genomic sequence only	587 (28%)	346 (31%)
T-DNA or Vector backbone	90 (4%)	N/A
Total	2,116 (100%)	1,110 (100%)

Table S2. Southern blot analysis of transformants

No. of T-DNA	No. of transformants
1	456 (82.3%)
2	73 (13.2%)
3	21 (3.8%)
4-6	4 (0.7%)
Total	544 (100%)

Table S3. Chromosomal distribution of TTLs

Chromosome	Length (Mb)	Phenotype-Defective Transformants			Randomly Selected Transformants		
		No. of observed TTLs	No. of expected TTLs[a]	*P*-value[b]	No. of observed TTLs	No. of expected TTLs[a]	*P*-value[b]
1	8.32	234	191	0.00*	42	31	0.05
2	6.33	181	145	0.00*	26	24	0.64
3	6.24	133	143	0.40	31	23	0.12
4	4.19	79	96	0.08	10	16	0.15
5	5.51	101	126	0.02*	15	21	0.21
6	4.64	125	106	0.07	17	17	0.93
7	4.38	72	100	0.00*	14	16	0.55
Unassinged	2.01	29	46	0.01*	1	8	0.02*
Total	41.62	954	954	-	156	156	-

[a] Expected numbers were calculated according to the chromosome length.

[b] χ^2-test is based on the difference between observed and expected values. Low *P*-values mean that they are significantly different from the expectation. χ^2 values are calculated as following function: $\chi^2=\Sigma\{(O-E)^2/E\}$. *P*-values are calculated with degree of freedom, 1.

* Significant at $P<0.05$

Table S4. Distribution of TTLs around genes

Type of region	Length (Kb)	Phenotype-Defective Transformants			Randomly Selected Transformants		
		No. of observed TTLs	No. of expected TTLs[a]	*P*-value[b]	No. of observed TTLs	No. of expected TTLs[a]	*P*-value[b]
Genic	31,954	704	732	0.29	95	120	0.02*
Coding region	19,888	229	456	0.00*	27	75	0.00*
Exon	16,937	175	388	0.00*	21	63	0.00*
Intron	2,651	54	61	0.39	6	10	0.21
5' promoter (< 1 Kb)	6,613	360	152	0.00*	55	25	0.00*
3' UTR (<0.5 Kb)	5,453	115	125	0.37	13	20	0.10
Intergenic	9,667	250	222	0.06	61	36	0.00*
Total	41,624	954	954		156	156	

[a] Expected numbers were calculated according to the chromosome length.

[b] χ^2-test is based on the difference between observed and expected values. Low *P*-values mean that they are significantly different from the expectation. χ^2 values are calculated as following function: $\chi^2=\Sigma\{(O-E)^2/E\}$. *P*-values are calculated with degree of freedom, 1.

* Significant at $P<0.05$

Table S5. Primer list used in TAIL-PCR

Name	Sequence[a] (5’ →3’)	Reference
LB1	GTCCGAGGGCAAAGAAATAGAGTA	-
LB2	CATGTGTTGAGCATATAAGAAACCCT	(Mullins et al. 2001)
LB3	GAATTAATTCGGCGTTAATTCAGT	(Mullins et al. 2001)
RB1	TTACAACGTCGTGACTGGGAAAAC	-
RB2	CTGGCGTAATAGCGAAGAGG	-
RB3	CCCTTCCCAACAGTTGCGCA	(Mullins et al. 2001)
AD1	NGTCGASWGANAWGAA	(Sessions et al. 2002)
AD-1	WAGTGNAGWANCANAGA	(Mullins et al. 2001)
AD2	TGWGNAGSANCASAGA	(Sessions et al. 2002)
AD3	AGWGNAGWANCAWAGG	(Sessions et al. 2002)
AD4	WAGTGNAGWANCANGAA	(Sessions et al. 2002)
AD6	WGTGNAGWANCANAGA	(Sessions et al. 2002)

[a] The degeneracy of AD primer sequence was followed the extended IUPAC code (A,T,G,C, W = A/T, S = G/C, N = A/T/G/C).
